# Insights into barriers and facilitators in PrEP uptake and use among migrant men and transwomen who have sex with men in Belgium

**DOI:** 10.1186/s12889-023-15540-y

**Published:** 2023-04-19

**Authors:** Ella Van Landeghem, Stef Dielen, Aline Semaan, Anke Rotsaert, Jef Vanhamel, Caroline Masquillier, Edwin Wouters, Kristien Wouters, Bea Vuylsteke, Thijs Reyniers, Christiana Nöstlinger

**Affiliations:** 1 Department of Public Health, Institute of Tropical Medicine Nationalestraat 155, 2000 Antwerp, Belgium; 2 Department of Sociology, University of Antwerp, Antwerp, Belgium; 3 Department of Clinical Sciences, Institute of Tropical Medicine, Antwerp, Belgium

**Keywords:** HIV prevention, PrEP, Access to health care, Migrants, Belgium

## Abstract

**Background:**

PrEP uptake is low among non-Belgian men and transwomen who have sex with men, although the HIV epidemic among men who have sex with men in Belgium is diversifying in terms of nationalities and ethnicity. We lack an in-depth understanding of this gap.

**Methods:**

We conducted a qualitative study using a grounded theory approach. The data consists of key informants interviews and in-depth interviews with migrant men or transwomen who have sex with men.

**Results:**

We identified four underlying determinants which shape our participants’ experiences and contextualize the barriers to PrEP use. These include (1) the intersectional identities of being migrant and men and transwomen who have sex with men, (2) migration related stressors, (3) mental health and (4) socio-economic vulnerability. Identified barriers include: the accessibility of services; availability of information, social resources and providers’ attitudes. These barriers influence PrEP acceptance and mediated by individual agency this influences their PrEP uptake.

**Conclusion:**

An interplay of several underlying determinants and barriers impacts on PrEP uptake among migrant men and transwomen who have sex with men, illustrating a social gradient in access to PrEP. We need equitable access to the full spectrum of HIV prevention and care for all priority populations, including undocumented migrants. We recommend social and structural conditions that foster exercising these rights, including adapting PrEP service delivery, mental health and social support.

## Introduction

Many countries have reported a concerning discrepancy between priority populations at risk for Human Immunodefiency Virus (HIV), and those being reached with HIV pre-exposure prophylaxis (PrEP), demonstrating a growing PrEP inequity, in particular among ethnic minorities [1–3]. This is also the case in Belgium, where PrEP is available and reimbursed through the national health system since 2017 (see Table 1). The Belgian HIV epidemic is characterized by two concentrated HIV epidemics, among men having sex with men, and non-Belgian heterosexuals. Against the background of an overall decrease in the number of new HIV infections reported, new cases among non-Belgians have become more diverse, seeing an increase among men who have sex with men from non-European origin, while new cases among Belgian men having sex with men were decreasing [4]. More specifically in 2020, 40% of men who have sex with men newly diagnosed with HIV had a Belgian nationality and 23% had another European nationality. This only includes men whose nationalities were known (i.e. 83% of all diagnoses among this group). This diversity is not reflected in PrEP uptake, considering that the majority of PrEP users are Belgian men who have sex with men (i.e. 75% of the PrEP starters in 2020). Clearly, the current number of migrants and ethnic minorities^1^ who are using PrEP does not reflect actual prevention need nor does it correspond to the ethnic composition of the newly reported HIV diagnosis [5]. Therefore, we need to better understand which barriers and facilitators migrant men and transwomen who have sex with men experience in accessing PrEP.

**Table 1:** PrEP service delivery in Belgium

PrEP is a biomedical HIV prevention method with particularly high efficacy demonstrated for men who have sex with men [6–8]. In Belgium, PrEP service delivery is centralized through specialized HIV clinics since 2017. PrEP is partly reimbursed under the national health insurance for individuals who meet the eligibility criteria [9] contingent on screening by a specialist physician. The user can then buy PrEP with an out-of-pocket cost of around €15 for 90 tablets [9, 10]. Belgium has a system of compulsory national health insurance for all registered residents, however, only legal residents have access to the health insurance scheme [11]. All asylum seekers are entitled to health care coverage while awaiting their asylum decision [12, 13]. Undocumented migrants are entitled to limited services through the system of urgent medical assistance (UMA). Because of an unclear definition of UMA, and a detailed and intrusive social enquiry conducted by the social welfare services at municipalities, the care people with undocumented status receive may differ [11].	

Few studies explored the factors accounting for the low uptake and use of PrEP among migrants within a European context. Some qualitative studies explored barriers on several socio-ecological levels across different contexts. These barriers may intersect and mutually influence and reinforce each other. At the individual level these factors include: lack of awareness and information [14–16] and low HIV risk perception [14]. Migrant men who have sex with men and people from Sub-Saharan African communities in Scotland reported concerns regarding adherence, and an anticipated negative impact on condom use [14]. At the community level, links to HIV stigma were reported as some people feared being identified as living with HIV, mistaking PrEP for HIV treatment, [14] and concerns about social exclusion due to involuntary PrEP disclosure to family members [2]. At the structural level several studies [15] indicated that current biomedical and centralized PrEP models may not be adapted to migrants’ needs. A Belgian community-based study with Afro-Latino-Caribbean men who have sex with men suggested a need for tailoring PrEP programmes towards this population, paying attention to the intersections between men who have sex with men and migrant communities [17]. Studies have also shown that once informed, migrant men and transwomen who have sex with men in several studies showed interest and potential acceptance of PrEP [16, 17]. There is a need for identifying additional pathways towards inclusive and equitable PrEP access to reach more diverse populations [18–23].

Five years into the PrEP roll-out in Belgium, we lack an in-depth understanding of why PrEP uptake and use remains low among migrants and ethnic minorities, in spite of their heightened vulnerability for HIV. Our main objective was to explore underlying determinants to PrEP acceptance and uptake and to investigate the factors and cross-cutting mechanisms that either hinder or facilitate PrEP uptake and use among migrant men and transwomen who have sex with men in Belgium.

## Methods

### Design

This inductive qualitative community-based study used a grounded theory approach [24], to be able to construct a theoretical explanatory framework uncovering the mechanisms of PrEP uptake, based on participants’ experiences and perception towards PrEP, and the meaning they attributed to it. We started with a formative research phase, collecting information among key informants to provide first insights from a higher level perspective. These data complemented and contextualized the core data, consisting of in-depth interviews with migrant men and transwoman who have sex with men.

### Study participants and recruitment

Key informants were selected based on their close contact with the target population, either as member of the community, a social worker or as health care provider. They were recruited through ongoing research collaborations with these organisations, existing HIV prevention networks and a multidisciplinary advisory board compiled for the study. Via snowball sampling we identified additional key informants.

For the in-depth interviews, we purposely selected portential participants with a known HIV risk (eg. at HIV testing facilities), but not yet using PrEP, as well as participants with PrEP use experience to contrast pathways. Recruitment venues were: two Belgian HIV clinics, sexual health organisations and social and medical organisations offering support to sex workers. We selected participants based on the following criteria: (1) being 18 years or above; (2) being currently on PrEP or having experience with PrEP, but not currently using it or being at risk of HIV infection but not a PrEP user; (3) having a migrant background (first or second generation), currently living in Belgium; (4) providing informed consent to voluntarily participate in the interview study; (5) speaking one of the languages in which the interviews could be conducted (Spanish, Arabic, Dutch, English or French). We deliberately kept our inclusion criteria broad, allowing for flexibility based on the direction the initial data collection showed us, in line with the use of theoretical sampling that is common in Grounded Theory approaches [24].

We recruited participants continuously in a similar manner: the recruiters – social workers or health care providers – identified suitable participants based on the inclusion criteria, and provided them with the relevant study information using multilingual flyers and information sheets. If potential participants showed interest, the recruiter asked verbal or written consent for being contacted by a researcher (first, second or third author). Upon consent, the researchers contacted participants and gave more extensive information about the study and asked to arrange for an interview. Participants were reimbursed for their time. Interviews were conducted in Dutch, French, English, Spanish or Arabic.

### Data collection

Data collection took place between October 2020 and June 2022, at a location convenient for the participant, such as the researcher’s office, in a park, café or at the participant’s personal home. There was no one else in immediate presence during the interview, unless the occasional exception of passers-by. We developed and used flexible, unstructured, and open-ended interview guides for both key informant and in-depth interviews. This interview guide was pilot-tested with other members of the research team. Topics included migration trajectories and personal context, HIV and sexually transmitted diseases (STI) risk perception and sexual health, motivations for whether or not using PrEP, decision-making process, trajectories in and experiences with PrEP use and anticipated or perceived barriers to PrEP use. Interviews were conducted face-to-face when possible. We used online technologies (Zoom) compliant with General Data Protection Regulation (GDPR) when COVID-19-restrictions prevented physical meetings with participants. Topic guides were developed in English, translated and conducted in the participant’s preferred language by the multilingual researchers (see research team and reflexivity). All participants consented to the interview being audio-recorded. Interview duration varied between 41 min and two hours. No repeat interviews were carried out.

### Data analysis

**Fig. 1:**
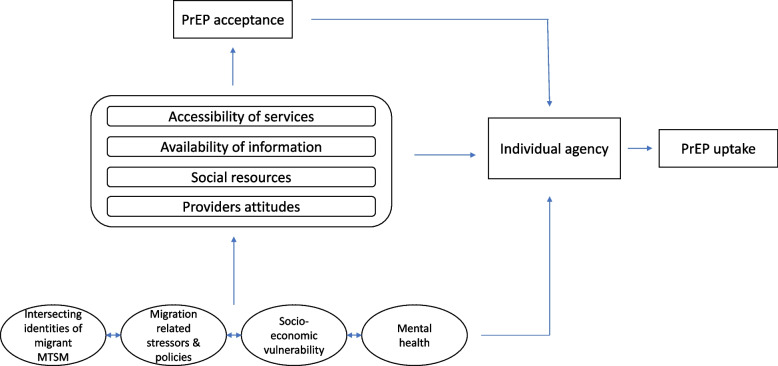
Explanatory conceptual framework

Audio recordings were transcribed verbatim in the interview language, translated to English when necessary and coded in NVivo 12. Initial coding was done by EVL. We analysed transcripts inductively, by applying a grounded theory approach [24, 25] using constant comparison methods across the collected data until data saturation was reached. The first analytical step included the preliminary analysis of notes and memos written during and after each interview. Then, we applied open coding to the raw data, focusing on identifying concepts that may determine PrEP uptake, and categorizing these concepts iteratively through constantly comparing initial with new codes to find consistencies and differences. Subsequently, we focused on finding patterns between the identified categories grounded in the data, refining theoretically relevant concepts. By analysing the linkages between these themes, we reached consensus among researchers and we developed a theoretical explanatory model (see Fig. 1). In a final step, all data sources were triangulated to reach a more comprehensive understanding and increase validity of the theoretical model.

### Research team and reflexivity

Dutch, English and French interviews were performed by EVL, Spanish by SD and Arabic by AS. At the time of data collection all reseachers were employed as pre-doctoral researchers at the Department of Public Health of the Institute of Tropical Medicine in Antwerp, Belgium. They are all holders of a Master’s degree in social or health sciences and are trained in qualitative research methods, with several years of experience. EVL and AS are female, SD is male. EVL and SD were born in Belgium, AS was born in Lebanon. No relationships were established prior to study commencement between participant and researchers. Researchers introduced themselves by their names, credentials, personal characteristics and interest in the study matter and population at the beginning of each interview.

## Results

### Participants

Table 2 presents the characteristics of 31 study participants. We conducted eight interviews with key informants and 23 interviews with migrant men or transwomen who have sex with men. Three key informants were recruited through our own networks, five were recruited through snowball sampling. Among the in-depth interview participants 14 were currently using PrEP; three were previous PrEP users, but did not use PrEP anymore at the time of the interview; and six participants had never used PrEP. All participants were born outside of Belgium, eight of them moved to Belgium less than four years ago, seven were living in Belgium between five and nine years, while eight participants had lived in Belgium for more than 10 years. Twelve participants were ever involved in transactional sex or sex work since arriving in Belgium. Sixteen participants were legal residents at the time of the interview, which includes full access to the Belgian health care system; but some had been living being undocumented for several years before that. Six participants had no legal residence status and one participant was an asylum seeker at the time of the interview. Data on refusal to participate was not collected to keep the administrative burden for recruiters as low as possible. One person who was identified as a potential participant by recruiters later refused because of distrust towards confidentiality and one person was lost to follow-up.

**Table 2:** Overview of study participants

**In-depth interviews (** ***n* = 23)**	
**Gender**	
Cisgender man	22
Transgender woman	1
**PrEP use**	
Current PrEP user	14
Never used PrEP	6
Former PrEP user	3
**Region of origin**	
North Africa	6
Middle East	6
Eastern Europe	1
Asia	3
Central- and South America	7
**Years in Belgium**	
0 to 4 years	8
5 to 9 years	7
Over 10 years	8
**Interview language**	
English	7
Spanish	6
French	6
Arabic	2
Dutch	2
**Residence status**^a^	
Documented	16
Undocumented	6
Currently in asylum procedure	1
**Ever involved in transactional sex or sex work**^a^	
Yes	12
No	11
**Key informant interviews (** ***n* = 8)**	
**Gender**	
Cisgender woman	5
Cisgender man	3
**Position/affiliation**	
Social worker	2
Nurse	4
Volunteer at community organization	2
**Language**	
Dutch	7
English	1

^a^Self-reported by respondents

### Codes and categories

Below, we present our findings in three parts. First, we discuss the underlying determinants for PrEP uptake. We define underlying determinants as social realities which influence barriers and facilitators and thus shape prevention behavior and PrEP uptake. In the second part we describe these barriers and facilitators, which are more related to environmental factors that hinder or facilitate PrEP uptake and use, however they must be understood within the context of the underlying determinants. The same factor can be both a barrier and a facilitator. Finally, we suggest a conceptual framework derived from the data explaining how these concepts are interrelated and influence the pathways to PrEP uptake and use (see Fig. 1). Although the conceptual definition of uptake and use differs, we mainly refer to PrEP uptake, unless otherwise stated. Not all participants are represented equally through quotes, these were selected to best demonstrate, contextualize or explain a particular finding. While these quotes are to a certain extent selective, the selection of themes is not and represents the main and balanced findings shared by all participants, as well as some relevant exemptions.

#### Underlying determinants of PrEP uptake

##### Intersecting identities: migrant and men and transwomen who have sex with men

*“How was my life in [country of birth]? (…) I’m gay, should I tell you about this life? Or my other personality? Which one do you prefer to hear about?”*

Participant 4 (PrEP-user).

Although not every participant self-identified as a gay man, many participants reported hiding their sexual orientation in their country of birth as a common experience, as the quote above illustrates. Participants reported coming from countries criminalizing same-sex behavior or from from strict heteronormative societies publicly rejecting gay people.

Most participants had left their home countries at least partially because of their sexual orientation. In some cases, participants’ family members had pressured them to leave once they learned about their sexual orientation. As one interviewee put it, gay men “*find a way out: leave the country or commit suicide*” (Participant 19, PrEP user). Additional reasons were unstable economic and political situations or professional or educational motivations. Generally, participants described their life in Belgium as liberating both in regard to their sexuality as well as living without any oppression or fear to be prosecuted.

*“That's the reason that, one of the reasons why I came here yeah, right. One of the biggest reasons there is, the freedom, the way to, the way to live fully, a little bit more openly homosexual”*

Participant 7 (former PrEP user).

Yet, some participants taking PrEP admitted that perceived sexual norms in the Belgian gay (e.g. through dating apps) had shaped their acculturation experience, which also influenced their PrEP uptake. This participant goes further to explain the role of PrEP in this integration process:

*“At the moment, you’re not part of this society. So we try to take any small step to improve ourselves to just be part of this society. (…) Any step that helps me as a new part of this society, to be more acceptable, be more successful, I will do it. And as a gay, taking PrEP is really a small step that I can take”*

Participant 4 (PrEP user).

##### Migration related stressors and restrictive policies

Life as a migrant in Belgium also presented other challenges. They perceived difficulties in understanding the country and its people, and navigating the health system. In general, many study participants mentioned experiences of racism and discrimination:

*“For example, when I was looking for apartment, they choose Belgian people. Or some girls in street, or something in the night, they see black hair, they move apart. They scared. I can understand it, but sometimes it’s a feeling of racism.”*

Participant 12 (never used PrEP).

Undocumented study participants feared being deported and thus often avoided much contact with the health care system. Otherwise, people who already had a long trajectory in social or migration services in Belgium often anticipated rejection, thus not trying to access healthcare services any longer:

*“As a rejected person, this is difficult for me. I had kidney stones and the ambulance did not take me to the hospital. You would have seen me lying on the road from the pain. (…) It was necessary to carry out the operation right away but they [reception centre] refused because it was an expensive operation. (…) I don’t give them [the Belgian healthcare system] 1 out of 10 [in terms of trust].”*

Participant 10 (never used PrEP).

Additionally, migration trajectories were impacted by migration policies in other countries, especially for transwomen:

*“Then the transsexuals started to travel, they started to travel to Europe, it was like a boom because in the past [region of origin] people wanted to immigrate to [country X]. Migration to [country X] was so difficult that without a visa, without documents or money you could do it. Then it was like another way to Europe opened up”*

Participant 2 (PrEP user).

##### Socio-economic vulnerability

Due to these restrictive migration policies, more than half of the participants found themselves in a precarious situation at least at some point in their life. Not being in the possession of legal documents in Belgium means having no direct access to health insurance, the formal job and housing market. Participants indicated having to struggle with many competing priorities on a day-to-day basis, making HIV prevention a lesser concern.

*“Look, I'll be honest, thank God I have food, I have a house, and I don't worry much, what worries me is having the money to pay for the house. That, and from there, nothing else.”*

Participant 11 (never used PrEP).

These socio-economic challenges led some of them to engage in transactional sex or sex work.

*“Because that's what I came to Europe to do, without papers, without anything, without speaking the language, without anything, what are you going to work on?”*

Participant 15 (PrEP user).

Most of our participants indicated to always use condoms with clients, but a few participants mentioned to be sometimes engaged in condomless sex in exchange of more money.

“*Sometimes they say: ‘if you can have sex without condom, I give you extra’. Sometimes I say yes.”*

Participant 12 (non-PrEP user).

##### Mental health

A number of participants explicitly referred to mental health as an important factor concerning sexual health and shared how mental health problems had influenced their individual agency in HIV prevention behavior:

*“Mental health is also important for sexual health (…) just because of the difficult situation I had, it conditions your mental state, it makes you commit more risks perhaps. Since in a normal state, you wouldn’t do certain things”*

Participant 7 (former PrEP user).

However, many participants also highlighted how the process of migration and integration may impact on their mental health in general:

"*So I lost myself self-confidence. All of us. The most important fact that they have to challenge with it, it’s the self-confidence. That they [migrants] lost it and they have to rebuild it. That’s the most important fact about migrants life*”

Participant 4 (PrEP user).

A few informants expressed concerns about substance abuse among their friends or social network in order to cope with these challenges. Some of them had experienced this themselves. These coping mechanisms were described as both a cause and a consequence of the migration related stressors, including loneliness. Indeed, some participants indicated having to take on sex work as a means of survival, while also having to resort to drugs or alcohol to be able to forget, thus staying in a vicious circle exacerbating their socio-economic and HIV vulnerability.

*“Belgium made me use more drugs. I felt myself alone and depressed. And I used drugs”*

Participant 12 (never used PrEP).

#### Barriers and facilitators to PrEP uptake and use

##### Accessibility of services

###### The role of residence status

One participant reflected on the problematic access to general health care when being undocumented:

*“I know that the health care system is very good because I have friends who have Belgian documents or [other European country] documents and they have had good care there. But with respect to us immigrants who do not have documents, ... it is difficult, even impossible to get to a doctor or a hospital or something like that.”*

Participant 14 (PrEP user).

Especially for PrEP uptake, the legal requirements to qualify for PrEP reimbursement were mentioned as the most substantial barrier for our participants, as PrEP can only be reimbursed for people who have health insurance coverage (see Table 1).

*“lf you don't have health insurance*,* you can't have PrEP. Because PrEP , I've heard from the pharmacy or something, is expensive. Is almost 100 euros or so. But if you have health insurance, you get that from 8 euros or 12 euros”*

Participant 9 (PrEP user).

The cost of PrEP without reimbursement even deterred some of them from trying to find out more information when they first had heard about PrEP, immediately classifying it as something beyond their reach.

*“I also heard from my friend that it's expensive, but condoms are free so I don’t see it as something for me”*

Participant 6 (never used PrEP).

If participants were able to access the Belgian healthcare system, they perceived the services received as very good, even though navigating and understanding the Belgian health care system was difficult.

###### High threshold of PrEP care

Once having accessed services, PrEP-users and key informants described how the process needed to get PrEP prescribed and reimbursed through specialized HIV clinics can be time-consuming.

*“The procedure simply takes far too long for our population. There are already waiting lists, often too long for them. Then you have to go, blood is taken, the doctor asks questions, you have to go to a nurse, there is a lot of information coming at you. Then you get a second appointment, a month later, but your PrEP still hasn't started, but in the meantime you're already about 3.5 months further on - and you're not on PrEP yet, so you have to get the explanation again because you've already forgotten what they said at the beginning, (...) So I think the procedure should be much faster if possible. I think that's definitely a threshold for them, and that for just the average middle-class gay man it's not a problem”*

Key informant D.

Especially the participants speaking mainly Arabic or Spanish (i.e. languages not commonly spoken in Belgium) mentioned language as one of the main problems in their everyday life and when accessing health care. This hindered knowledge and information seeking, making appointments, communicating with health care providers and being able to express concerns and ask questions to them.

*“I do not know much about other diseases, even when the doctor asked me to do several tests, I agreed without knowing them except for AIDS because there was some difficulty in communicating because of the language (...) I mean, the last thing was she did not understand me and I did not understand her. She even got to the point of translating via computer and show me what she meant”*

Participant 10 (never used PrEP).

In addition, some key informants alluded to the perceived association of HIV clinics with homosexuality or clients’ assumed promiscuous behavior. Some participants feared that they might be seen as HIV positive when visiting an HIV clinic, which they assumed might pose an extra barrier for PrEP uptake for others.

*“It [location] is very much known as an HIV clinic. Um. [They think] that everyone who comes here is HIV-positive. Really, the gossip… Sometimes you even see people coming for [something else]. But they have their mouth mask on up to here and cap. Just because they don't want to be seen. Because they are afraid that people might gossip about them, or 'say, I saw that one in the [location] and that one might have [HIV]”*

Key informant G.

However, other PrEP-users reported having had no problems in accessing PrEP services, and they also felt reassured by the medical screening. These were mostly PrEP-users with an established life in Belgium. They had legal documentation, were employed, spoke either English, Dutch or French and had a social network in Belgium in combination with a high risk perception.

*“When I moved to [city], the [HIV clinic], it is so well located. It seems so clean. I made an appointment very easily. I could have an appointment I think 5 days after I searched online”*

Participant 13 (PrEP user).

Three of our participants were former PrEP users. They had stopped using PrEP because of experienced side-effects or because it interfered with drugs taken for building muscles. One participant was forced to stop PrEP because he only informally had access (see also: alternative pathways to PrEP) and informal access often got interrupted due to low availability of PrEP at the community-based organisation.

###### Alternative pathways to PrEP: theory and practice

Some community organisations provided an alternative pathway to receiving PrEP outside the regular facility-based delivery. Four of our participants accessed PrEP informally through community-based organisations. In this cases, medical professionals prescribed PrEP and provided follow-up, but free of charge for the users. However, many key informants mentioned that this was an unsustainable system, as the organisations did not always have enough PrEP in stock to answer the growing demand.

##### Availability of information

Information received by our participants about sexual health and HIV in particular were strongly influenced by whether or not they had received sexual education in their country of birth, their use of the internet, their social networks or through their contact with lesbian, gay, bisexual and transgender (LGBT) organisations or activists in their country of birth. In general, participants perceived their knowledge about HIV prevention to be quite high, however, some key informants expressed concerns about others. As one of them commented:

*“For example in some countries, homosexuality is never discussed. Let alone sexual health or STIs, they don't know what that is. So often with those clients it's just starting from 0. (…) then PrEP is already quite complicated”*

Key informant B.

Many participants shared that people in their environment still did not know enough about HIV prevention and that PrEP was not widely promoted. Key informants confirmed this, and indicated how they struggled to explain PrEP when stigma, myths and misconceptions about HIV were dominating the discourse. Especially stories about the side effects of PrEP were reported to circulate: many times this was the first thing a participant shared about what they knew of PrEP. PrEP-users were often influenced by friends who already took PrEP and shared positive experiences, while other participants (both users and non-users) indicated existing misconceptions, which could also shy people away from PrEP. The following key informant provided a nunaced understanding of how the persisting fear of side-effects can be linked to HIV stigma and the rejection of HIV risk:

*“There are those who don't want to take PrEP or don't want to take their HIV medication because that confirms to them every time that they are taking a risk, or that they are HIV-positive. That they're not mentally ready for that yet and they don't want to think about that. Yes, denial and then ignoring out of fear”*

Key informant D.

As illustrated by the quotes below, social networks, including social media, functioned as an important source of knowledge, and influenced attitudes and perceptions about PrEP. While some participants believed it was nearly impossible for a gay man in Belgium not to know about PrEP, others did not fully agree. The latter perceived PrEP still as something that was happening on the margin and knowledge should be better disseminated, especially among undocumented migrants.

*“Maybe it's because when you come here for free and without papers, people are afraid, people don't trust anyone. So instead of asking for help, they are afraid to talk, they always go with insecurity because they always go with insecurity that the police or things like that, so sometimes that can happen”*

Participant 16 (PrEP user).

##### Social resources

We identified diverse meanings of social networks: it referred to a few close people, a community organisation, one family member or even a neighbour. The lack of a social network often led to feeling lonely, which negatively affected mental health on the longer term.

*“I’m alone, I feel alone. I just have separated with my boyfriend and I don’t have any family here and I cannot find a friend or find someone for a relationship, so. It’s a little bit difficult for me”*

Participant 1 (PrEP user).

For participants who had some social resources, this could also be protective against the negative mental health effects or to support in overcoming structural difficulties, such as finding a job or a place to live.

*"My boss from my volunteer work helped me. Because she knows my situation and I explained everything. She has a friend who lives here in the city, has a big house and has a studio in the roof. She asked me, if you want, I have a friend and you can live there”*

Participant 9 (PrEP user).

##### Providers’ attitudes

The way participants were received by their health care provider for the first time played a significant role in their attitudes towards uptake of PrEP, and in general health care seeking alike. Many indicated the importance of feeling understood, to be received in a respectful manner, and to have the opportunity to ask questions. Participants who had a negative experience at their first encounter, noted that this could scare people away from seeking necessary services.

*“The first time I was here, I was paranoia, I am afraid of HIV, I am afraid of the PrEP and so on. I was with an old doctor who does not laugh and explained everything very dryly. That was a bit difficult for me”*

Participant 9 (PrEP user).

#### PrEP acceptance

A belief in effectiveness, the effect it had on reducing worries about potential HIV acquisition, and perceiving PrEP as a responsible choice all underpinned PrEP acceptance and uptake within our study population.

*“I felt, when I started the PrEP I felt more relaxed (…) because for chlamydia, for gonorrhea or syphilis there will always be an injection or pills, but for HIV there is no injection or pills”.*

Participant 16 (PrEP user).

Participants’ with a lower PrEP acceptance explained this mainly stemmed from not being convinced about its effectiveness. They indicated a need to see some sort of proof that it works. Key informants attributed this skepticism to a lack of real understanding of PrEP grounded in a general distrust in medical information. This often led to people finding their own explanations.

*“I just don’t find it very credible that it really protects you. There is no physical barrier, you cannot see it. While with condoms you clearly can see it. I would need real proof to know if it works”*

Participant 5 (never used PrEP).

However, for some PrEP users, using PrEP meant taking care of their sexual health. This reduced their worries about acquiring HIV, and made their sexual experiences more care-free and pleasurable.

*“Exactly, it has given me the freedom to enjoy my sexual life, which I never did before. Because I was always scared, you know”*

Participant 3 (PrEP-user).

#### Individual agency in prevention behavior

PrEP users shared a great belief in PrEP effectiveness and a high motivation to use PrEP, in particular those with high risk perception. Having lived through years of fear of getting HIV, some even described themselves as hypochondriacs or health maniacs. They often saw PrEP as a responsible choice, indicating that striving for protecting their health encouraged them to go on PrEP.

*“Well the truth is, I think they [PrEP users] are smart on their part because if there is a pill that helps you do that function of protecting your body, why not take it?”*

Participant 15 (PrEP user).

Furthermore, even if their environment was not always safe or predictable, most participants using PrEP had a feeling of control over their sexual health, enabling them to protect themselves.

*“Yes it worries me, but at the end of the day in this job [sex work] it’s like Russian roulette. (...) and you have to take care of yourself as far as you can. If it happens to you, well, it's destiny and you have to take your treatment (...) because if you are working as an escort, as a prostitute, you know that it can happen, that is, at any moment it may not be HIV but it can be syphilis, or other sexually transmitted diseases, you know what I mean, so you have to have a clear mind that if you don’t take care of yourself it could happen”*

Participant 14 (PrEP user).

Additionally, although asylum centers do not formally include PrEP within the health care they provide (see Table 1), actual practices might differ contingent on individual agency of either supportive health care providers, or proactive asylum-seekers. As as one participant indicated: *“I just asked, it was never a problem”* (Participant 21, PrEP user).

However, for some, maintaining their health status functioned rather as an argument against PrEP. They perceived PrEP – and medication in general – as something intoxicating the body, thus avoided taking medications when it is not for treating an illness, and opted for condoms as a preventive measure.

*“There is nothing wrong with me, why would I take pills? I am sexual, I protect myself. But why would I take pills?”*

Participant 8 (never used PrEP).

Finally, while some participants were generally accepting towards PrEP, it did not lead directly to PrEP uptake because it did not match their current prevention needs.

*"I tell others if you do a lot of sex with a lot of people, 100% you have to take it, but in my case right now, I don't do clients"*

Participant 6 (never used PrEP).

### Pathways towards PrEP: building an explanatory conceptual framework

As shown above, we identified several concepts that influenced the pathway towards PrEP for migrant men and transwomen who have sex with men (see Fig. 1). Participants described underlying determinants and challenges associated with being a migrant and belonging to a sexual minority. These intersecting identities and corresponding circumstances influenced both mental health and socio-economic vulnerability, two other important underlying determinants of their PrEP uptake. These determinants shaped participants’ everyday life. We identified four barriers and facilitators to PrEP acceptance. Firstly, the accessibility of PrEP services, tied to legislative and organisational barriers. Currently, PrEP service delivery is embedded within a high threshold sexual health care system, which could not always accommodate the complex needs of our participants. Information availability and social resources could function as both a barrier and facilitator. Finally, providers’ attitudes towards our participants were another factor impacting on PrEP acceptance and thus influencing PrEP uptake. Additionally, PrEP acceptance influenced PrEP uptake through individual agency: it could function as a way to overcome barriers and lead to PrEP uptake despite many limiting factors.

## Discussion

This study’s main objective was to acquire an in-depth understanding of PrEP uptake and use among migrant men and transwomen who have sex with men. The results, brought together in the conceptual explanatory framework (see Fig. 1), are consistent with the few studies available, demonstrating that low PrEP uptake among migrants might be related to structural barriers and vulnerabilities and competing social and health priorities [14, 15]. The data clearly explain the high HIV vulnerability for structurally disadvantaged migrant men and transwomen who have sex with men, which paradoxically does not translate in an increasing PrEP uptake due to administrative and legislative barriers on a system level. Indeed, similar to what other researchers have found, the data show a mismatch between the health care utilization and the profile of the people who would benefit most from it [21]. In addition, our findings confirm the major influence of minority stressors, stemming both from being a migrant and belonging to a sexual minority group, on PrEP acceptance, uptake and use [26, 27]. For undocumented migrants, access to health care in general remains a difficult issue. Even though there are systems in place, they are too complicated to navigate for both health care providers and potential users, and thus remain under-utilized [28–30]. Additionally, many participants engaged in sex work out of necessity, mainly driven by the structural vulnerabilities they experienced [31] which could have an impact on their sexual health. Alternative pathways to PrEP for migrants have been developed through bottom-up adaptive change, such as community based organisations providing PrEP outside of the formal system, although they may not be sustainable on the long term. The available studies also emphasized the importance of correct knowledge about PrEP [14–16, 32, 33]. This was confirmed by our participants.

Some of our findings are not reported elsewhere. Although some studies highlighted PrEP stigma and difficulties to disclose PrEP use, [15] this phenomenon was only reported in regard to visiting the healthcare facility, and not towards family members. This might be explained by the fact that many were forced to migrate because of their sexual orientation. Arriving in Belgium, they often did not have a large network or family they should hide their PrEP use from. On the contrary, PrEP use was seen as a way of integration in the Belgian men and transwomen who have sex with men communities, as it was normalized on dating apps and through word of mouth. Consequently, we may conclude that PrEP acceptance depends on the social group of reference or community of initial acculturation [34], as PrEP could symbolize a membership of a certain social group or network [35]. The undocumented status of some of our participants, which made them officially ineligible for PrEP reimbursement, directly led to a higher HIV vulnerability through for instance survival sex work due to economic insecurity. Nevertheless, we also saw differences in access to PrEP depending on migration pathways, where voluntary migrants reported less barriers than involuntary migrants. This may have also have had an impact on their mental health and social resources, as found in other studies [36].

Mental health problems have been well-documented among sexual minorities [37–40] and migrants [41–43], most often caused by minority stress, discrimination and racism. Our data also shows that that participants’ intersecting identities led to a high mental health burden. Consistent with other studies [34, 41, 44, 45] sexual minority experiences in the country of birth and socio-economic adversity in the country of residence influenced sexual agency and HIV prevention behavior including PrEP uptake. Community connectedness and social support [34] are often mentioned in the literature as protective factors, which is also in line with our results. Our results thus confirm the important role of mental health in HIV prevention and add to the call for integrating HIV prevention and mental health support [46].

This study has several limitations: firstly, since we recruited mainly in a clinical context, study participants were aware of, or already in contact with, PrEP. Additionally, non-users were underrepresented in this study, because of difficulties in identifying participants who would be theoretically eligible for PrEP. Thus, we may have missed out on perceptions of people who are not yet reached by social or health services, implicating that there may be other barriers than those documented here. Secondly, despite efforts to recruit a diverse study population through purposive sampling, Sub-Saharan African migrants were not represented in this study. We did not succeed in recruiting this group firstly because only very few Sub-Saharan African migrants were using PrEP in Belgium at the time of the study, [5] while at the same time recruiters experienced difficulties in convincing this specific population to participate. Additionally, the in-depth interview with our transwoman participant did not suggest any major differences in barriers towards PrEP compared to our cis-male participants. However, since we did not include more than one transwoman, this makes it more difficult to provide an in-depth comparison between men and transwomen among our participants. Finally, parts of our data were collected while different COVID-19 restrictions were in place. Recruitment was slower than anticipated and collecting data online posed several challenges, potentially influencing rapport building, and the richness of the responses. We may also have missed out on non-verbal cues through body language.

Future research could address some of these limitations. From a public health perspective, it is necessary to contextualize PrEP within a wider frame of migrant health inequalities, and it is important to invest in social services and outreach workers and to effectively link social with health services, such as PrEP navigators in the USA [47], peer-led education programmes in the UK [48] or key population community health workers in Thailand [49]. Since we found mental health strongly linked to participants’ social and economic situation, we suggest to integrate social support into PrEP care for those living in precarious circumstances. Furthermore, PrEP should be promoted and made accessible, physically and financially in a sustainable and equitable way, for everyone irrespective of legal residence status using an inclusive approach. On an organisational health care level, there is a need for interpreters, as well as multi-lingual, culturally and structurally competent health care staff. At the intersection of health care provision and community-based prevention, a shift in PrEP programmes, their promotion messages and language used will be required [50]. Promoting PrEP as a health promotion rather than as risk reduction tool could improve its acceptance and uptake, as it would disconnect PrEP use from the social stigma related to being an HIV drug [51]. Changes on a structural and policy level are required by simplifying social administration procedures and decentralising HIV and PrEP services [52]. Our findings also stress the need for an inclusive migration policy, preventing structurally forcing people in precarious and undocumented positions [53].

## Conclusion

Men and transwomen who have sex with men in Belgium experience multi-level barriers and facilitators when accessing PrEP, driven by their intersecting identities, migration-related stressors impacting on mental health and socio-economic conditions. Factors shaping access at different socio-ecological levels mediate this access including availability of information, social resources, accessibility and provider-related factors. Their interplay determines individual HIV prevention agency leading to PrEP uptake and use. Our findings thus identified a social gradient in access to PrEP: not all migrant men and transwomen who have sex with men were equally vulnerable, suggesting less of a cultural but more of a structural influence on these issues. These results suggest that, in general, PrEP is deemed acceptable yet not always accessible to men and transwomen who have sex with men. To reach UNAIDS 95–95-95 by 2030 [54], access to the full spectrum of HIV prevention should be granted to everyone, regardless of residence status. Improving the social and structural circumstances in which these rights can be exercised is key, along with strengthening mental health support and building of social resources.

## Data Availability

The data presented in this article are not readily publicly available because they contain information that could compromise the privacy of our research participants. A list of condensed meaning units or codes could be made available upon reasonable request to the corresponding author.

## Abbreviations

PrEP: Pre-exposure Prophylaxis
HIV: Human Immunodefiency Virus
UMA: Urgent Medical Assistance
STI: Sexually Transmitted Diseases
GDPR: General Data Protection Regulation
LGBT: Lesbian, gay, bisexual and transgender

## Footnotes

1 We use the “migrant and ethnic minorities” as a term to describe anyone who was not born in Belgium or Western-Europe. We are aware that this is a very diverse population and can include (non-exhaustive list): asylum-seekers, refugees, documented migrant workers and people with irregular status, as well as people with different lengths of residence/stay in the country.
